# Serum Lutein and Zeaxanthin Are Inversely Associated with High-Sensitivity C-Reactive Protein in Non-Smokers: The Mikkabi Study

**DOI:** 10.3390/antiox11020259

**Published:** 2022-01-28

**Authors:** Mieko Nakamura, Minoru Sugiura

**Affiliations:** 1 Department of Community Health and Preventive Medicine, Hamamatsu University School of Medicine, Hamamatsu 431-3192, Japan; 2 Department of Food Science and Nutrition, Faculty of Human Life and Science, Doshisha Women’s College of Liberal Arts, Kyoto 602-0893, Japan; msugiura@dwc.doshisha.ac.jp

**Keywords:** carotenoid, xanthophyll, antioxidant, inflammation, smoking

## Abstract

Recent randomized controlled trials have demonstrated a protective association between carotenoids and inflammation; however, the basis of this association on lifestyle factors remains unclear. This study aimed to clarify the associations between carotenoids and inflammatory markers stratified by lifestyle factors, using baseline data from the Mikkabi Study. Serum carotenoid and high-sensitivity C-reactive protein (hs-CRP) levels were measured. Multivariable adjusted odds ratios (ORs) for a high hs-CRP level (≥2.0 mg/dL) were obtained using logistic regression analysis. The data of 882 individuals were analyzed; 11.7% had high hs-CRP levels. The highest tertile of lutein (OR: 0.44; 95% confidence interval [CI]: 0.25–0.76), zeaxanthin (OR: 0.36; 95% CI: 0.21–0.64), total carotenoid (OR: 0.57; 95% CI: 0.32–0.9997), and oxygenated carotenoid concentration (OR: 0.50; 95% CI: 0.28–0.90), with the lowest tertile as reference, was inversely associated with a high hs-CRP level. The interaction between lutein, but not other carotenoids, and current smoking was significant. The inverse association between lutein and a high hs-CRP level was significant in non-smokers (OR: 0.41; 95% CI: 0.22–0.76) but not in smokers. These results further support the anti-inflammatory effect of carotenoids; nevertheless, further studies should clarify the interaction of smoking with the association between lutein and inflammation.

## 1. Introduction

To establish normal homeostasis, acute inflammation activated by microbial or viral infections, exposure to allergens or toxins, or trauma is usually protective to the host; however, chronic inflammation is harmful to the body and increases the risk of cellular damage, resulting in the development of chronic diseases, including cardiovascular disease, autoimmune disease, neurological disease, and cancer [1,2]. C-reactive protein (CRP) has been widely used as an acute-phase inflammatory marker, and a high-sensitivity CRP (hs-CRP) assay has been developed to detect a slight increase within the normal range, which reflects persistent systemic low-grade inflammation and is considered to be one of the risk factors for many chronic diseases [3,4,5]. Indeed, various epidemiological studies have revealed that increased CRP levels are associated with all-cause mortality, cardiovascular mortality, cancer mortality, mental disorders, and physical frailty in older people [5,6,7,8].

Carotenoids that are abundant in fruits and vegetables have been suggested, in experimental and epidemiological studies, to have anti-inflammatory properties in addition to antioxidant and antiapoptotic properties [9,10,11,12]. During inflammation, immune system-related cells—including macrophages and leukocytes—are evoked at the site of damage, resulting in oxidative stress via the overproduction of reactive oxygen species; carotenoids can act directly or indirectly against such oxidative stress [1]. A recent meta-analysis of randomized controlled trials (RCTs) revealed a protective effect of carotenoids against inflammation [12]. However, these RCTs are not sufficient to show an association between carotenoids and inflammation based on lifestyle factors such as smoking, alcohol intake, and exercise.

Therefore, we aimed to clarify the association between carotenoids and inflammatory markers stratified by lifestyle factors, using data from a population-based study.

## 2. Materials and Methods

### 2.1. The Mikkabi Study and Participants

This study was conducted using baseline data from the Mikkabi Study (cohort I). A detailed study protocol has been reported previously [13,14,15]. Briefly, the study participants comprised inhabitants who received an annual health check-up conducted by the local government of Mikkabi town, Shizuoka, Japan. In total, 302 men and 584 women aged between 30 and 70 years were enrolled between April and May 2003. Among these, in this report, we analyzed the data for 299 men and 583 women whose serum carotenoid and hs-CRP levels were examined.

### 2.2. Measurements

For the health check-up, blood samples were obtained in the morning after the participants had fasted overnight. Serum was separated from blood cells by centrifugation, and stored at −80 °C until the serum carotenoid assessment was conducted. After storage for no longer than 1 year, the concentrations of six serum carotenoids (α-carotene, β-carotene, β-cryptoxanthin, lutein, lycopene, and zeaxanthin) were assessed using a reverse-phase high-performance liquid chromatography (HPLC) system at the Laboratory of Public Health and Environmental Chemistry, Kyoto Biseibutsu Kenkyusho, Kyoto, Japan. First, the serum samples were mixed with H_2_O and ethanol containing β-apo-8′-carotenal and extracted into hexane. The organic layer was removed, evaporated to dryness at room temperature, resolved in chloroform:ethanol (1:19), and transferred to a microvial for automatic injection. The concentrations were monitored at 480 nm. The HPLC was fitted with a 201TP54 reverse-phase C18 column, and β-apo-8′-carotenal was used as an internal standard. The mobile phase was composed of methanol:tetrahydrofuran:H_2_O (94:5:1), and the flow rate of the phase was 0.8 mL/min. The peaks of the six carotenoids were identified based on the retention time and quantified using standard curves of authentic carotenoids. Further details of the serum carotenoid assessments can be found elsewhere [13,14,15]. For the method used in the study, the detection limit for the serum lycopene concentration was 0.04 µg/mL (0.075 µmol/L), and values below this limit were recorded as 0.03 µg/mL (0.056 µmol/L) in the subsequent analysis. The total carotenoid, oxygenated carotenoid (i.e., xanthophylls), and hydrocarbon carotenoid (i.e., carotenes) levels were obtained from the sum of the six carotenoids; the sum of β-cryptoxanthin, lutein, and zeaxanthin; and the sum of α-carotene, β-carotene, and lycopene, respectively. Serum hs-CRP was measured using a latex nephelometry immunoassay method (N-Latex CRP II; Dade Behring Holdings, Inc., IL, USA). All blood measurements, apart from those of serum carotenoid and hs-CRP levels, were conducted at the laboratory of Seirei Preventive Health Care Center, Shizuoka, Japan.

Height, body weight, and blood pressure were measured by trained staff. Body mass index (BMI) was calculated as body weight divided by height squared (kg/m^2^). Blood pressure was measured using an automated sphygmomanometer (Model BP-103iII; Nihon Colin, Inc., Aichi, Japan).

Information on current smoking, regular alcohol intake (≥once per week), and habitual exercise (weekly participation) was obtained using a self-administered questionnaire, which was reviewed by the investigators. The total energy intake was assessed using a validated simple food frequency questionnaire developed especially for the Japanese population [16,17]. Our previous study showed that the serum carotenoid concentration was significantly associated with dietary carotenoid intake; it was significantly low in participants with smoking and alcohol intake [18]. Thus, smoking and alcohol consumption were the main lifestyle factors focused on in the present study.

### 2.3. Statistical Analyses

Participants were divided into high and low hs-CRP groups (≥2.0 mg/L and <2.0 mg/L, respectively) according to the cut-off value used in the Jupiter study [19]. Serum carotenoid, hemoglobin A1c, and triglyceride levels that were skewed toward the higher concentrations were natural log-transformed to improve the normality of their distribution in the applicable analysis, and the results were back-transformed to their original scale in the table.

The characteristics of the participants in the high and low hs-CRP groups are described as mean (standard deviation [SD]) for continuous variables or number (percentage) for categorical variables; they were compared using *t*-tests for continuous variables and chi-square tests for categorical variables. Odds ratios (ORs) and 95% confidence intervals (CIs) for high hs-CRP levels were obtained with the tertiles of serum carotenoids for the whole cohort, using the lowest tertile as reference. Age and sex were adjusted for in Model 1, and BMI, current smoking, regular alcohol intake, habitual exercise, and total energy intake were further adjusted for in Model 2. In addition, ORs and 95% CIs for high hs-CRP levels were obtained using serum carotenoid concentration as a continuous variable, with adjustment variables used as in Model 2. Furthermore, stratified analyses for age (<50 years and ≥50 years), sex, BMI (<25 kg/m^2^ and ≥25 kg/m^2^), current smoking (yes/no), regular alcohol intake (yes/no), and habitual exercise (yes/no) were further conducted to obtain ORs and 95% CIs for high hs-CRP levels adjusted for age, sex, BMI, current smoking, regular alcohol intake, habitual exercise, and total energy intake, excluding the relevant variable used in the stratification.

Data were analyzed using IBM SPSS Statistics for Windows, Version 28.0 (IBM Corp., Armonk, NY, USA). All tests were two-tailed, and statistical significance was set at *p* < 0.05.

## 3. Results

### 3.1. Participant Characteristics

The data from a total of 882 individuals were analyzed, and 11.7% showed high hs-CRP levels (≥2.0 mg/dL). A summary of participant demographic characteristics, lifestyle factors, and physical and blood examination findings, stratified according to hs-CRP levels, are presented in Table 1. Participants with higher hs-CRP levels were more likely to be men, with a higher BMI, higher triglyceride and hemoglobin A1c levels, increased white blood cell count, and a lower high-density lipoprotein cholesterol (HDL-C) level. Regarding serum carotenoids, lutein, zeaxanthin, β-carotene, and lycopene concentrations were low in participants with high hs-CRP levels. Total and hydrocarbon carotenoid concentrations were also significantly lower in participants with high hs-CRP levels.

### 3.2. Association between Serum Carotenoid and High-Sensitivity C-Reactive Protein Levels

Table 2 presents the ORs and 95% CIs for the high hs-CRP level according to tertiles of serum carotenoid concentration, with the lowest tertile as the reference, adjusted for age and sex (Model 1) and additionally adjusted for BMI, current smoking, regular alcohol intake, habitual exercise, and total energy intake (Model 2). The highest tertile of lutein (OR: 0.44; 95% CI: 0.25–0.76), zeaxanthin (OR: 0.36; 95% CI: 0.21–0.64), total carotenoid (OR: 0.57; 95% CI: 0.32–0.9997), and oxygenated carotenoid (OR: 0.50; 95% CI: 0.28–0.90) concentrations were associated with a high hs-CRP level in Model 2.

**Figure 1:**
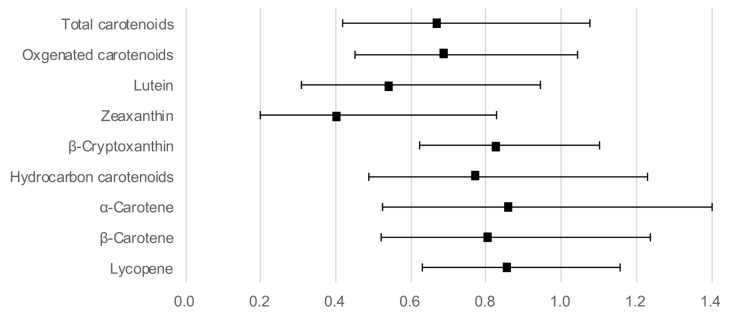
Odds ratios and 95% confidence intervals for a high hs-CRP level with serum carotenoid concentrations as continuous variables, adjusted for age, sex, body mass index, current smoking, regular alcohol intake, habitual exercise, and total energy intake.

Figure 1 shows the ORs and 95% CIs for a high hs-CRP level with serum carotenoids as continuous variables, adjusted for age, sex, BMI, current smoking, regular alcohol intake, habitual exercise, and total energy intake. Lutein (OR: 0.54; 95% CI: 0.31–0.94) and zeaxanthin (OR: 0.41; 95% CI: 0.20–0.83) concentrations showed a significant inverse association with a high hs-CRP level. In addition, the association between the other four carotenoids and a high hs-CRP level did not reach statistical significance, although the OR was below 1.

**Figure 2:**
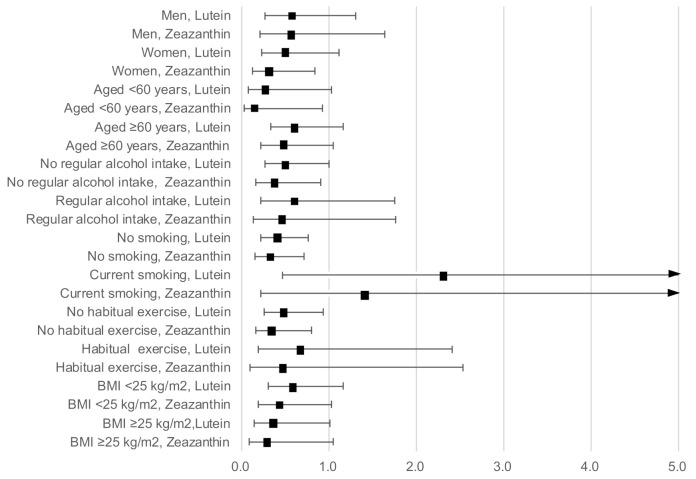
Odds ratios and 95% confidence intervals for a high hs-CRP level with lutein and zeaxanthin concentrations as continuous variables stratified by sex, age, BMI, current smoking, regular alcohol intake, and habitual exercise, adjusted for age, sex, BMI, current smoking, regular alcohol intake, habitual exercise, and total energy intake (excluding the relevant variable used in the stratification).

Figure 2 shows the results of the stratified analysis presenting ORs and 95% CIs for a high hs-CRP level with serum lutein and zeaxanthin concentration as continuous variables, adjusted for age, sex, BMI, current smoking, regular alcohol intake, habitual exercise, and total energy intake (excluding the relevant variable used in the stratification). The inverse associations of a high hs-CRP level with lutein and zeaxanthin concentration remained stable across sexes, individuals of younger and older ages, regular alcohol drinkers and non-drinkers, those who undertook regular exercise and those who did not, and those with a high and a low BMI.

However, current smoking and lutein concentration showed significant interactions (*p* = 0.02). Serum lutein concentration was significantly and inversely associated with a high hs-CRP level in non-smokers (OR: 0.41; 95% CI: 0.22–0.76), but not in smokers (OR: 2.32; 95% CI: 0.46–11.64). The interaction between smoking and zeaxanthin concentration was not significant (*p* = 0.14), although a similar contradictory association was observed between non-smokers (OR: 0.32; 95% CI: 0.15–0.71) and smokers (OR: 1.43; 95% CI: 0.21–9.51).

The interaction between other carotenoids and current smoking was insignificant (α-carotene, *p* = 0.19; β-carotene, *p* = 0.64; β-cryptoxanthin, *p* = 0.67; and lycopene, *p* = 0.48).

## 4. Discussion

In this observational study, we tried to clarify the associations between individual carotenoids and inflammatory markers stratified by lifestyle factors such as smoking, alcohol intake, and exercise. Serum carotenoid concentration showed a broadly protective association with systemic low-grade inflammation (represented by hs-CRP); this association was more evident for serum lutein and zeaxanthin. Notably, an inverse association between serum lutein and hs-CRP levels was observed exclusively in non-smokers.

The protective association between carotenoids and inflammation is broadly in line with findings from previous observational [9,20] and intervention studies [12]. Hozawa et al., have reported that serum total carotenoids are inversely associated with CRP in U.S. young adults [9]. In a cross-sectional study, van Herpen-Broekmans et al., demonstrated that the inverse association between serum carotenoid and hs-CRP levels was significant only for β-carotene and vitamin C [20]. However, a meta-analysis of RCTs revealed that lutein/zeaxanthin and β-cryptoxanthin—that is, oxygenated carotenoids—were inversely and significantly associated with CRP [12]. The results of the present study further support the anti-inflammatory effects of lutein and zeaxanthin.

We cannot elucidate why only lutein and zeaxanthin showed a significant protective association with hs-CRP, although it is hypothesized that the structural features of these carotenoids may play related anti-inflammatory roles in the membrane of multiple bilayer systems such as phospholipid bilayers [10,11]. Lutein and its structural isomer zeaxanthin, as well as other carotenoids, have a skeleton composed of 40 carbon atoms organized into eight isoprene units that are responsible for nonpolar (i.e., hydrophobic) regions [10,21]. Both lutein and zeaxanthin, as well as β-cryptoxanthin, are categorized as oxygen-containing xanthophylls, owing to their cyclic hexenyl structure with an attached hydroxyl group responsible for polar (i.e., hydrophilic) regions at the ends of carbon–carbon bonds [10,21]. Although β-cryptoxanthin has a hydroxyl group at one end of the carbon chain, lutein and zeaxanthin have hydroxyl groups at both ends of the carbon chain. Thus, in the phospholipid bilayer of membranes, lutein and zeaxanthin can align within the membrane region by binding each hydroxyl group to each of the double membrane surfaces (polar region) and placing a carbon chain in the membrane core (non-polar region); however, non-polar hydrocarbon carotenoids can only be located in the membrane core [10]. 

In the present study, the inverse association between serum lutein and hs-CRP levels was only significant in non-smokers, not in smokers. In smokers, high serum lutein levels tend to increase the risk of low-grade inflammation. Watzl et al., reported a significant CRP reduction via a high intake (eight servings per day) of carotenoid-rich vegetables and fruit in non-smoking men [22]. Wang et al., reported a significant CRP reduction via 20 mg lutein-supplementation in healthy non-smoking men and women [23]. In a pilot intervention study, Graydon et al., reported no apparent change in CRP levels associated with foods rich in both lutein and zeaxanthin (spinach powder) or lutein and zeaxanthin supplements in healthy volunteers, including current smokers [24]. In an RCT, Estevez-Santiago et al., also reported no significant change in CRP levels after daily supplementation of 6 mg lutein and 2 mg zeaxanthin in healthy female participants; however, there was no information on smoking status [25]. Nevertheless, Riso et al., observed by post hoc analysis that 10 day supplementation with broccoli (each portion [250 g] provided 3.1 mg of lutein, 1.4 mg of β-carotene, and 146 mg of vitamin C) decreased CRP levels in young healthy smokers [26]. In our previous study, smoking was not associated with serum lutein or zeaxanthin concentrations [18]. Whether smoking influences the association between lutein and CRP should be further investigated. However, planned intervention studies employing lutein supplementation to assess this association in smokers should be considered with caution.

The present study had several limitations. The cross-sectional nature of the study design limits the inference of causality in the observed association. The number of people included in the stratified analysis may not have been large enough, especially for smokers; thus, the insignificantly higher OR between lutein and a high hs-CRP level in smokers could have been caused by chance. Finally, although we controlled for potential confounding factors in the statistical model, there is a possibility that other factors might have still confounded the results.

In conclusion, the findings of the present study—in addition to those of previous observational and intervention studies—further support the anti-inflammatory effects of carotenoids. An inverse association between carotenoid and CRP levels was more evident for lutein and zeaxanthin, while a protective association between lutein and CRP levels was confirmed exclusively in non-smokers. Further studies should be conducted to clarify the interaction between smoking, lutein, and hs-CRP to establish the protective effect of lutein on persistent systemic low-grade inflammation.

## Figures and Tables

**Table 1:** Summary of participant demographic characteristics, lifestyle factors, and physical and blood examination findings stratified according to high-sensitivity C-reactive protein level.

Characteristics	hs-CRP < 2.0 mg/L		hs-CRP ≥ 2.0 mg/L		*p*-Value ^1^
	*n* = 779		*n* = 103		
Age, years, mean (SD)	55.4	(9.8)	56.3	(10.8)	0.36
Men, *n* (%)	255	(85.3)	44	(14.7)	0.04
Women, *n* (%)	524	(89.9)	59	(10.1)	
Current smoking: no, *n* (%)	690	(88.5)	90	(11.5)	0.72
Current smoking: yes, *n* (%)	89	(87.3)	13	(12.7)	
Regular alcohol intake: no, *n* (%)	586	(88.9)	73	(11.1)	0.34
Regular alcohol intake: yes, *n* (%)	193	(86.5)	30	(13.5)	
Habitual exercise: no, *n* (%)	591	(88.1)	80	(11.9)	0.75
Habitual exercise: yes, *n* (%)	184	(88.9)	23	(11.1)	
Body mass index, kg/m^2^, mean (SD)	22.7	(2.8)	24.8	(4.2)	<0.01
Systolic blood pressure, mmHg, mean (SD)	130	(20)	134	(18)	0.10
Diastolic blood pressure, mmHg, mean (SD)	77	(12)	79	(11)	0.09
Total cholesterol, mg/dL, mean (SD)	212	(34)	213	(36)	0.63
LDL cholesterol, mg/dL, mean (SD)	126	(31)	130	(31)	0.21
HDL cholesterol, mg/dL, mean (SD)	65	(17)	60	(16)	<0.01
Triglycerides, mg/dL, mean ^2^	90		102		0.04
Hemoglobin A1c, %, mean ^2^	5.1		5.4		<0.01
White blood cell count, mean ^2^	5287		6064		<0.01
Total energy intake, kcal, mean (SD)	2079	(589)	2109	(543)	0.62
Total serum carotenoids, µmol/L, mean ^2^	3.48		3.11		0.03
Oxygenated carotenoids, µmol/L, mean ^2^	2.36		2.12		0.08
Lutein, µmol/L, mean ^2^	0.57		0.52		0.01
Zeaxanthin, µmol/L, mean ^2^	0.24		0.22		0.01
β-Cryptoxanthin, µmol/L, mean ^2^	1.37		1.23		0.23
Hydrocarbon carotenoids, µmol/L, mean ^2^	1.03		0.89		0.01
α-Carotene, µmol/L, mean ^2^	0.13		0.12		0.07
β-Carotene, µmol/L, mean ^2^	0.59		0.51		0.02
Lycopene, µmol/L, mean ^2^	0.26		0.22		0.04

hs-CRP: high-sensitivity C-reactive protein; SD: standard deviation; LDL: low-density lipoprotein; HDL: high-density lipoprotein. ^1^ *p*-value for differences were obtained using *t*-tests for continuous variables and chi-square tests for categorical variables. ^2^ Means were calculated using log-transformed values and shown as the original scale.

**Table 2:** Adjusted odds ratios and 95% confidence intervals for a high high-sensitivity C-reactive protein level according to tertiles of serum carotenoid concentration with the lowest tertile of each carotenoid as a reference using logistic regression analysis.

Serum Carotenoids		Tertiles of Serum Carotenoid Concentration ^1^				
		T1 (Low)	T2		T3 (High)	
		OR	OR	95% CI	OR	95% CI
Total serum carotenoids	Model 1 ^2^	1.00	0.56	(0.33–0.93)	0.53	(0.30–0.91)
	Model 2 ^3^	1.00	0.52	(0.30–0.90)	0.57	(0.32–0.9997)
Oxygenated carotenoids	Model 1 ^2^	1.00	0.58	(0.35–0.98)	0.50	(0.29–0.86)
	Model 2 ^3^	1.00	0.58	(0.34–0.99)	0.50	(0.28–0.90)
Lutein	Model 1 ^2^	1.00	0.78	(0.49–1.26)	0.40	(0.23–0.70)
	Model 2 ^3^	1.00	0.81	(0.50–1.33)	0.44	(0.25–0.76)
Zeaxanthin	Model 1 ^2^	1.00	0.59	(0.36–0.97)	0.37	(0.21–0.64)
	Model 2 ^3^	1.00	0.56	(0.34–0.94)	0.36	(0.21–0.64)
β-Cryptoxanthin	Model 1 ^2^	1.00	1.07	(0.64–1.81)	0.79	(0.45–1.39)
	Model 2 ^3^	1.00	0.97	(0.56–1.68)	0.74	(0.41–1.34)
Hydrocarbon carotenoids	Model 1^2^	1.00	0.80	(0.49–1.31)	0.53	(0.30–0.95)
	Model 2 ^3^	1.00	0.87	(0.52–1.44)	0.68	(0.37–1.25)
α-Carotene	Model 1 ^2^	1.00	0.75	(0.46–1.22)	0.56	(0.31–1.001)
	Model 2 ^3^	1.00	0.78	(0.47–1.30)	0.64	(0.35–1.18)
β-Carotene	Model 1 ^2^	1.00	0.72	(0.43–1.20)	0.56	(0.31–1.005)
	Model 2 ^3^	1.00	0.79	(0.46–1.34)	0.74	(0.40–1.37)
Lycopene	Model 1 ^2^	1.00	0.64	(0.39–1.06)	0.59	(0.34–0.997)
	Model 2 ^3^	1.00	0.61	(0.36–1.02)	0.63	(0.36–1.08)

OR: odds ratio; CI: confidence interval. ^1^ Tertiles of serum carotenoid concentration (µmol/L): Total carotenoids, ≥4.27, 2.75–4.27, <2.75; Oxygenated carotenoids, ≥2.96, 1.74–2.96, <1.74; Lutein, ≥0.65, 0.47–0.65, <0.47; Zeaxanthin, ≥0.26, 0.19–0.26, <0.19; β-Cryptoxanthin, ≥2.10, 0.94–2.10, <0.94; Hydrocarbon carotenoids, ≥1.38, 0.84–1.38, <0.84; α-Carotene, ≥0.15, 0.11–0.15, <0.11; β-Carotene, ≥0.82, 0.47–0.82, <0.47; Lycopene, ≥0.39, 0.20–0.39, <0.20. ^2^ Adjusted for age and sex. ^3^ Adjusted for age, sex, body mass index, current smoking, regular alcohol intake, habitual exercise, and total energy intake.

## Data Availability

The data are not publicly available due to that informed consent and ethical approval were not included to open the data.
